# Supplementary material for: Shigella OspF blocks rapid p38-dependent priming of the NAIP–NLRC4 inflammasome
Source: Proc Natl Acad Sci U S A. 2026 Jan 14;123(3):e2510950123. doi: 10.1073/pnas.2510950123 (PMC12818434; doi:10.1073/pnas.2510950123)
Supplement: Supplementary file 1 — Appendix 01 (PDF) [file pnas.2510950123.sapp.pdf]

PNAS

Supporting Information for
Shigella OspF blocks rapid p38-dependent priming of the NAIP–NLRC4 inflammasome

Elizabeth A. Turcotte[a], Kyungsub Kim[b,c], Kevin D. Eislmayr[a], Lisa Goers[b], Patrick S. Mitchell[d,e], Cammie F. Lesser[b,c,f,g,1], Russell E. Vance[a,h,i,j,1]

[1]To whom correspondence may be addressed. Email: cammie.lesser@tufts.edu or rvance@berkeley.edu.

This PDF file includes:

Figures S1 to S7
Tables S1 to S3
Supporting Materials and Methods

Supporting Figures and Tables

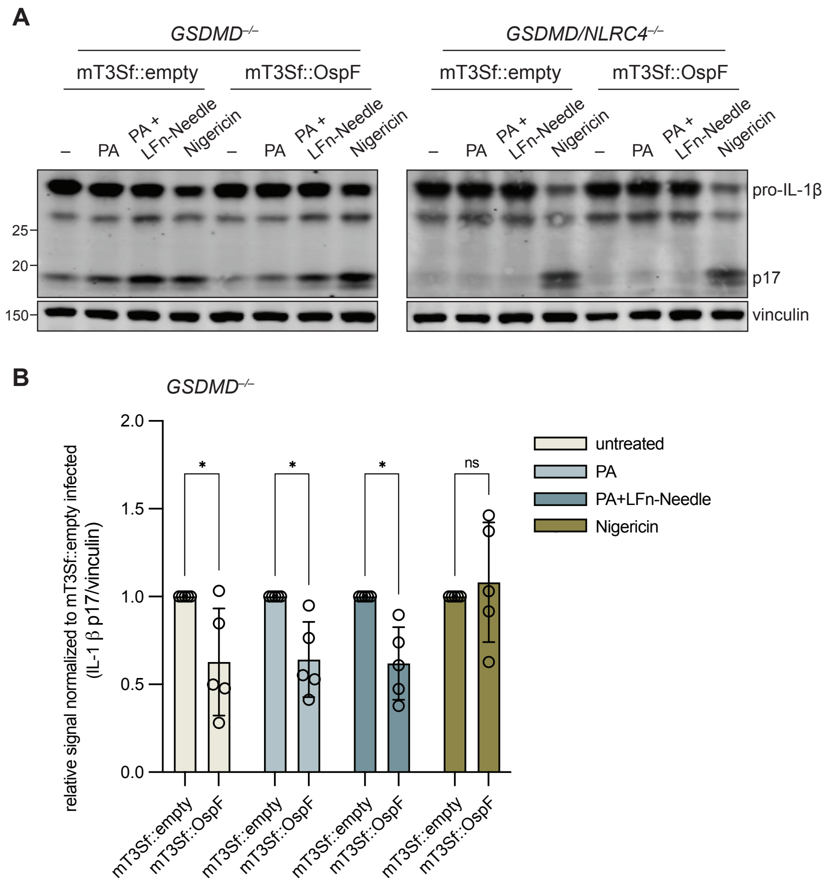

Figure S1. mT3Sf::OspF infection suppresses NLRC4-dependent IL-1β processing. Western Blot of lysates from *GSDMD*$^{-/-}$ or *GSDMD/NLRC4*$^{-/-}$ (**A**) and quantification (**B**) of A of *GSDMD*$^{-/-}$ THP-1 cells infected at MOI 5 with mT3Sf::empty or mT3Sf::OspF for 1 h before challenge with PA, PA + 100 ng/mL LFn-Needle, Nigericin, or left unchallenged for 2 h. Data are representative of five independent experiments. Quantification represents p17 band signal normalized by vinculin, and shown relative to mT3Sf::empty-infected band for each challenge condition. Data represent the mean ± SD. Two-way ANOVA. *$^{*}P < 0.0332$, $^{**}P < 0.0021$, $^{***}P < 0.0002$, $^{****}P < 0.0001$.*

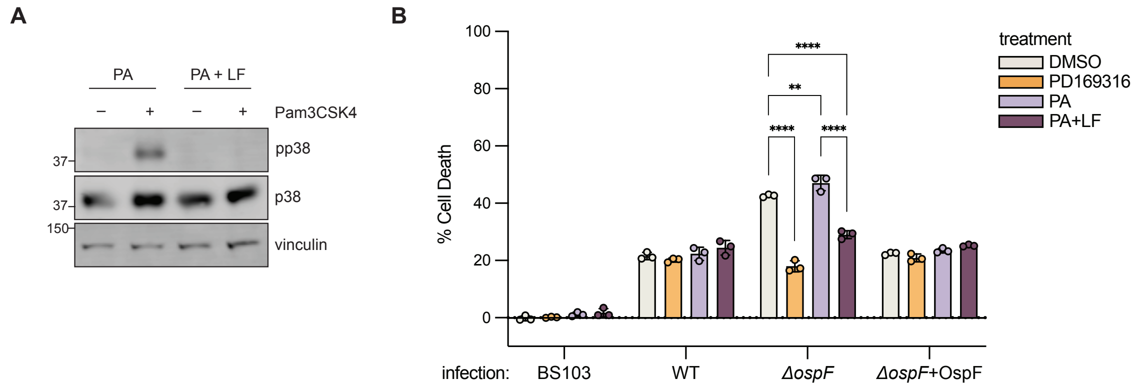

Figure S2. Lethal Factor inactivation of MKKs suppresses NLRC4-dependent cell death during *ΔospF* infection. A. Western Blot of lysates from WT THP-1 cells treated with PA or PA + Lethal Factor (LF) for 1 h before priming with Pam3CSK4 for 1 h. **B.** WT THP-1 cells treated pre-treated for 1 h with DMSO, PD169316, PA, or PA + LF before infection with *Shigella* at an MOI of 10. Data shown are from one experiment, which are representative of more than three independent experiments. Individual data points represent technical replicates. Cell death was measured at 1 hpi by PI uptake and calculated as % Cell Death relative to TritonX-100 treatment. Data represent the mean ± SD. Two-way ANOVA. **P* < *0.0332, **P* < *0.0021, ***P* < *0.0002*, *****P* < *0.0001* (A-B, D-E).

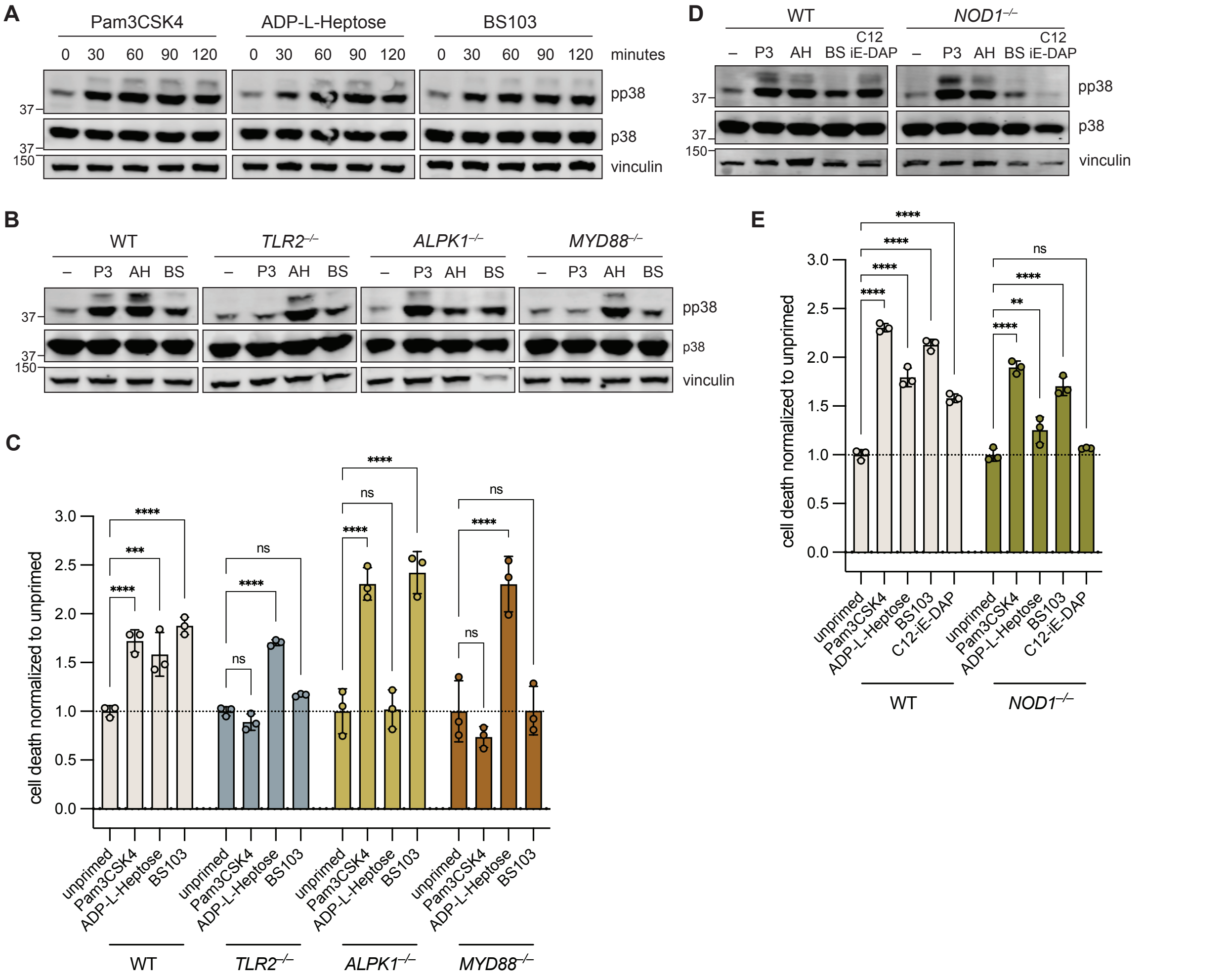

Figure S3. TLR2, ALPK1, and NOD1 activation can prime NAIP–NLRC4. A. Western Blot of lysates from priming timecourse of WT THP-1 treated with Pam3CSK4, ADP-L-Heptose, or BS103. **B.** and **C.** THP-1 knockout validation of *TLR2*, *ALPK1*, and *MYD88*$^{-/-}$ THP-1 cells for priming induced phospho-p38 (pp38) (B), and enhanced response to NeedleTox challenge (C). **D.** and **E.** THP-1 knockout validation of *NOD1*$^{-/-}$ THP-1s for priming induced pp38 (D), and enhanced response to NeedleTox challenge (E). C and E Cell death was measured at 3 hpc by PI uptake and calculated as % Cell Death relative to TritonX-100 treatment. Fold change of cell death of primed cells relative to unprimed cells, all treated with NeedleTox. Data shown are from one experiment, which are representative of more than three independent experiments. Individual data points represent technical replicates. Data represent the mean ± SD. Two-way ANOVA. *$^{*}P < 0.0332$, $^{**}P < 0.0021$, $^{***}P < 0.0002$, $^{****}P < 0.0001$.*

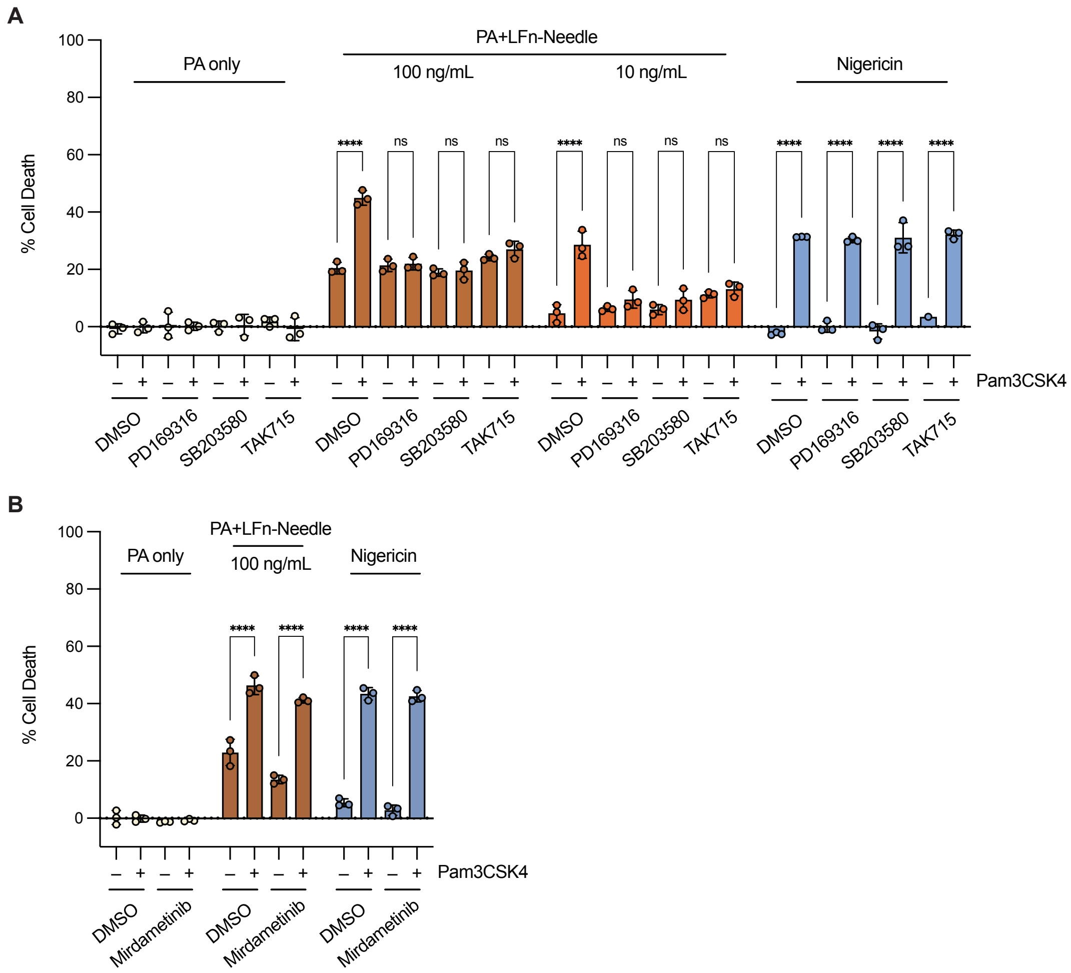

Figure S4. p38 inhibitors suppress rapid priming of NAIP–NLRC4. A. WT THP-1 cells pre-treated for 1 h with DMSO or p38 inhibitors, PD169316, SB203580, or TAK715 before challenge with PA, PA+LFn-Needle, or Nigericin. **B.** WT THP-1 cells pre-treated for 1 h with DMSO or MEK1/2 inhibitor Mirdametinib before challenge with PA, PA+LFn-Needle, or Nigericin. Data shown are from one experiment, which are representative of more than three independent experiments. Individual data points represent technical replicates. Cell death was measured at 3 hpc by PI uptake and calculated as % Cell Death relative to TritonX-100 treatment. Data represent the mean ± SD. Two-way ANOVA. **P < 0.0332, **P < 0.0021, ***P < 0.0002*, *****P < 0.0001.*

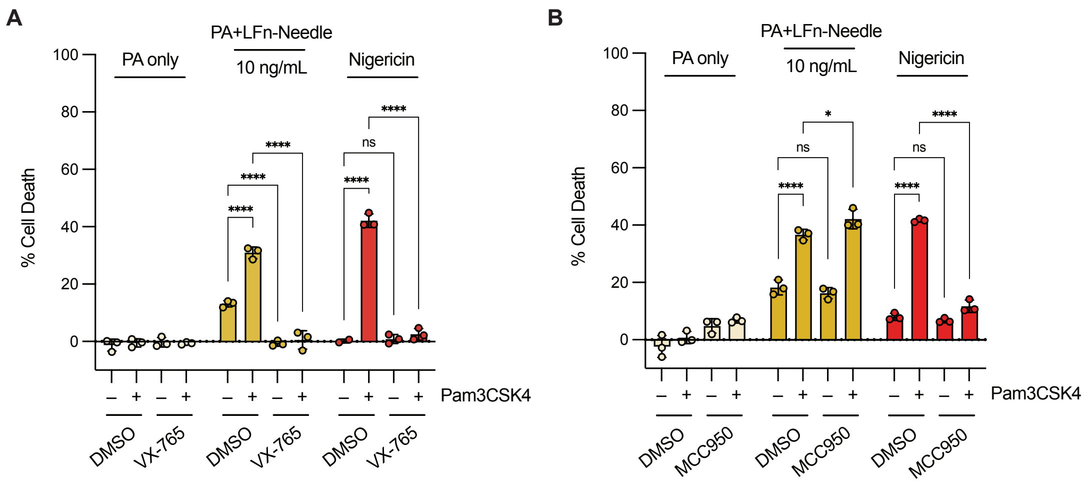

Figure S5. Priming of NAIP–NLRC4 is CASP1-dependent and NLRP3-independent. A. WT THP-1 cells pre-treated for 1 h with DMSO or 20 μM Caspase-1 inhibitor VX-765 before challenge with PA, PA+LFn-Needle, or Nigericin. **B.** WT THP-1 cells pre-treated for 1 h with DMSO or 10 μM NLRP3 inhibitor MCC950 before Pam3CSK4 prime for 1 h and challenge with PA, PA+LFn-Needle, or Nigericin. Data shown are from one experiment, which are representative of more than three independent experiments. Individual data points represent technical replicates. Cell death was measured at 3 hpi by PI uptake and calculated as % Cell Death relative to TritonX-100 treatment. Data represent the mean ± SD. Two-way ANOVA. **P < 0.0332, **P < 0.0021, ***P < 0.0002*, *****P < 0.0001* (A).

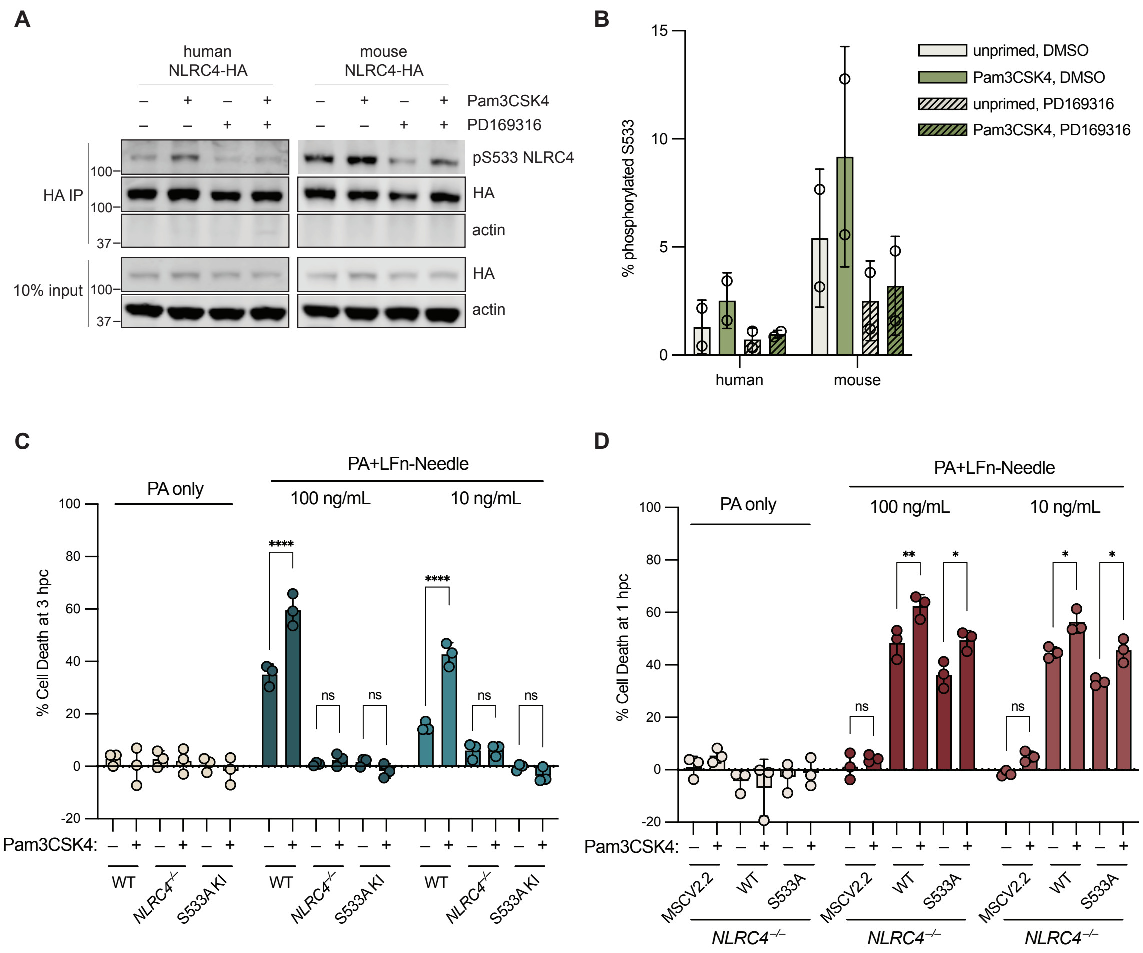

Figure S6. Phosphorylation of NLRC4 S533 is regulated by p38 but is not required for rapid priming. A. Western Blot of HA IP of NLRC4-HA in *GSDMD*$^{-/-}$ THP-1 cells upon DMSO or 5 µM PD169316 treatment for 1 h before prime with Pam3CSK4 for 1 h. **B.** Quantification of % pS533 NLRC4. Phosphorylated S533 band/total HA-tagged NLRC4. **C.** WT, *NLRC4*$^{-/-}$, or S533A knock-in (KI) THP-1 cells were primed for 1 h with Pam3CSK4 before challenge with PA only or NeedleTox. **D.** Sorted THP-1 cells with low expression of MSCV2.2 empty or MSCV2.2 NLRC4^{WT} or NLRC4^{S533A} were primed for 1 h with Pam3CSK4 before challenge with PA only or NeedleTox. Data shown are from one experiment, which are representative of more than three independent experiments. Individual data points represent technical replicates (C and D). Cell death was measured at 3 hpc (**C**) or 1 hpc (**D**) by PI uptake and calculated as % Cell Death relative to TritonX-100 treatment. Data represent the mean ± SD. Two-way ANOVA. **P < 0.0332, **P < 0.0021, ***P < 0.0002*, *****P < 0.0001.*

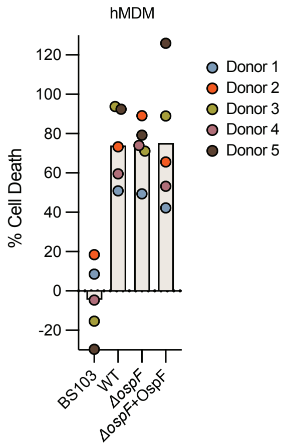

Figure S7. Cell death during primary HMDM infection is not suppressed by OspF. Primary HMDMs infected with *Shigella flexneri* (MOI 5). Each point represents an individual donor (the mean of three technical replicates). Cell death was measured at 1 hpi by PI uptake and calculated as % Cell Death relative to TritonX-100 treatment.

Table S1. Bacterial strains used in this study

Name	Description	Source
**Shigella flexneri (WT)**	*S. flexneri* 2457T	
**BS103**	*S. flexneri* 2457T virulence plasmid cured	(*84*)
**∆*ospF***	*S. flexneri ∆ospF*	(*24*)
**∆*ospF*+OspF**	*S. flexneri ∆ospF* + pAM238-OspF	(*24*)
**∆*ospF*+KLA**	*S. flexneri ∆ospF* + pAM238-OspF KLA	this study
**∆*ospF*+SpvC**	*S. flexneri ∆ospF* + pAM238-SpvC	this study
**mT3Sf**	BS103 *ipa/mxi(∆mxiE)/spa* + pNG162 VirB	this study
**mT3Sf::empty**	mT3Sf + pDSW206-empty	this study
**mT3Sf::ospB**	mT3Sf + pDSW206-OspB	this study
**mT3Sf::ospC1**	mT3Sf + pDSW206-OspC1	this study
**mT3Sf::ospC2**	mT3Sf + pDSW206-OspC2	(*26*)
**mT3Sf::ospC3**	mT3Sf + pDSW206-OspC3	(*26*)
**mT3Sf::ospD1**	mT3Sf + pDSW206-OspD1	this study
**mT3Sf::ospD2**	mT3Sf + pDSW206-OspD2	this study
**mT3Sf::ospD3**	mT3Sf + pDSW206-OspD3	this study
**mT3Sf::ospE2**	mT3Sf + pDSW206-OspE2	this study
**mT3Sf::ospF**	mT3Sf + pDSW206-OspF	this study
**mT3Sf::ospG**	mT3Sf + pDSW206-OspG	this study
**mT3Sf::ospI**	mT3Sf + pDSW206-OspI	this study
**mT3Sf::ospZ**	mT3Sf + pDSW206-OspZ	this study
**mT3Sf::ipaH1.4**	mT3Sf + pDSW206-IpaH1.4	this study
**mT3Sf::ipaH4.5**	mT3Sf + pDSW206-IpaH4.5	this study
**mT3Sf::ipaH7.8**	mT3Sf + pDSW206-IpaH7.8	this study
**mT3Sf::ipaH9.8**	mT3Sf + pDSW206-IpaH9.8	this study
**mT3Sf::ipaJ**	mT3Sf + pDSW206-IpaJ	this study
**mT3Sf::ipgB2**	mT3Sf + pDSW206-IpgB2	this study
**mT3Sf::virA**	mT3Sf + pDSW206-VirA	this study

Table S2. gRNA target sites used in this study

Gene target	Sequence
NLRC4	5′-ATCGTGTGAGCAGTGATGGATGG-3′
CASP4	5′-GCCACTGAAAGATACATACGTGG-3′
GSDMD	5′-GCATGGGGTCGGCCTTTGAGCGG-3′
NAIP	5′-ACATTGCCAAGTACGACATAAGG-3′
ASC	5′-ACCGGGCTGCGCTTATCGCGAGG-3′
TLR2	5′-TGGAAACGTTAACAATCCGGAGG-3′
MYD88	5′-GGTTGAGCTTACCTGGAGAGAGG-3′
ALPK1	5′-GTTGGAAGCGCCAGATGTGTCGG-3′
NOD1	5′-ATCTCAACGACTACGGCGTGCGG-3′
$NLRC4^{S533A}$ KI	5′-CAAGAGGCCTCTCTGGAGACAGG-3′

Table S3. Primary antibodies used in this study

Target	Catalog	Dilution
**IL-1β**	R+D, MAB201	1:1000
**vinculin**	CST, #13901	1:1000
**phospho-p38 MAPK**	CST, #9211	1:1000
**p38 MAPK**	CST, #9212	1:1000
**phospho-ERK1/2 MAPK**	CST, #4370	1:1000
**ERK1/2 MAPK**	CST, #4695	1:1000
**HA**	Roche, 11867423001	1:1000
**phospho-S533 (NLRC4)**	ECM Biosciences, NP5411	1:1000
**β-actin**	Santa Cruz Biotechnology, sc-47778	1:1000

Supporting Materials and Methods

Immunoprecipitation

Treated cells expressing NLRC4-HA constructs were collected by cell lifter in cold PBS. Cells were lysed in 30 mM Tris-HCl, pH7.5, 150 mL NaCl with 0.5% Nonidet P40, with HALT protease and phosphatase inhibitors for 1 h on ice. Lysates were centrifuged at max speed for 20 min. Supernatant was incubated with Pierce anti-HA magnetic beads for 3 h at 4°C, rotating. After washing, samples were eluted in 1x Laemmli buffer at 70°C for 10 min. Samples were then used for Western Blot as described in methods.

CRISPR-Cas9 Generation of THP-1 Knock-in Cell Line

For generation of knock-in $NLRC4^{S533A}$, THP-1 cells were nucleofected with Alt-R™ S.p. Cas9 Nuclease V3 (IDT) complexed with sgRNA, and Alt-R™ Cas9 Electroporation Enhancer (IDT). Before combining with cells, IDT Alt-R™ HDR donor oligo was added to Cas9-sgRNA complexes. Cells were nucleofected in Lonza P3 buffer (Lonza, V4XP-3032) using the Lonza 4D Nucleofector Core Unit, program CM-137. Nucleofected cells were recovered in media containing 1 μM IDT Alt-R™ HDR Enhancer V2 (10007910) for 18 h. Media was then changed to remove enhancer. Cells were plated for monoclones. Knock-in efficiency was sequenced and analyzed by ICE analysis (Edit Co). Guide and donor sequences were designed using Alt-R™ CRISPR HDR Design Tool (IDT). ssODN HDR oligo sequence: 5′-TCAGAATTTCTTGCTCAGTGGTGTTTTTCACACTTTGCAAGGCTTCCTGCCGCCAGAGAGGCCTCTTGGCGATGGAAAGTCCGAGAAGGCA-3′.

Guides used are listed in Table S2.
